# Investigation on the cellular mechanism of Prunetin evidenced through next generation sequencing and bioinformatic approaches against gastric cancer

**DOI:** 10.1038/s41598-022-15826-y

**Published:** 2022-07-13

**Authors:** Preethi Vetrivel, Santhi Nachimuthu, Abusaliya Abuyaseer, Pritam Bhagwan Bhosale, Sang Eun Ha, Hun Hwan Kim, Min Young Park, Gon Sup Kim

**Affiliations:** 1 Research Institute of Life science and College of Veterinary Medicine, Gyeongsang National University, Gajwa, Jinju, 52828 Republic of Korea; 2 Department of Pharmacy, National University of Singapore, Singapore, 119077 Singapore; 3 Department of Biochemistry, Biotechnology and Bioinformatics, Avinashilingam Institute for Home Science and Higher Education for Women, Coimbatore, 641043 India

**Keywords:** Gastrointestinal cancer, Genomics, Sequencing

## Abstract

Gastric cancer is the common type of malignancy positioned at second in mortality rate causing burden worldwide with increasing treatment options. More accurate and reliable diagnostic methods/biomarkers are urgently needed. The application of transcriptomics technologies possesses the high efficiency of identifying key metabolic pathways and functional genes in cancer research. In this study, we performed a transcriptome analysis on Prunetin treated AGS cells. A total of 1,118 differentially expressed (DE) genes on Prunetin treated AGS cancer cells, among which 463 were up-regulated and 655 were down-regulated. Notably, around 40 genes were found to be related with necroptosis, among which 16 genes were found to be in close association with Receptor Interacting Protein Kinase (RIPK) family. Validation of the RIPK genes through GEPIA identified 8 genes (*NRP1, MNX1, SSRP1, PRDX2, PLRG1, LGALS4, SNX5* and *FXYD3*) which are highly expressed in stomach cancer were significantly down-regulated in PRU treated samples. In conclusion, the sequencing data explores the expression of RIPK mediated genes through necroptosis signaling network in treating gastric cancer. The futuristic validations on the 8 genes as candidate biomarkers will offer a treatment approach against gastric cancer using PRU.

## Introduction

Despite improvements in survival rates over the last few decades, GC has only been diagnosed at an advanced stage, with very poor prognoses and the highest chance of recurrence^1^. GC is biologically and genetically heterogeneous, with a poor understanding of molecular carcinogenesis^2^. Biomarkers are distinct features that can be objectively measured and analyzed as indicators of biological processes, pathogenic mechanisms and pharmacological responses to a therapeutic intervention^3^. Despite the fact that numerous studies on GC biomarkers have been published, the majority of the identified targets fail in validation studies^4^. Patients in advanced stages are still unable to be treated with targeted therapy, and there are currently no diagnostic markers available. The altered regulation of gene expression is a major reason for tumors to express different cancer biomarkers. As a result, significant progress has been made in identifying the key mediators of GC at the molecular level^5^.

Chemotherapeutics are currently at a higher risk of failure due to their high toxicity. As a result, numerous studies are being conducted to investigate the use of natural compounds as potential drug candidates^6,7^. Prunetin (PRU) is an O-methylated flavonoid extracted from various plant sources that has numerous biological activities such as anti-obesity, anti-inflammatory, and proteolytic activity^8^. Targeting pathways associated with malignancy and progression would be a novel strategy for understanding the progression of GC and its detailed genomic characterization^9^.

Transcriptome sequencing is a rapidly evolving technique that provides an unprecedented view of the transcriptome profile^10^. It is now widely accepted that the introduction of high-throughput sequencing technology has accelerated the analysis of tumor behavior at the molecular level^11^. Identification of transcriptome sequencing using RNA-seq yields a large amount of information on expressed sequence tags (ESTs) and the discovery of new functional genes, which is beneficial to the development of molecular markers. Furthermore, RNA-seq technology, which has been widely used in disease diagnosis, pharmaceutical mechanism, and drug screening, could be used to assess the spatial and temporal gene expression of specific cell genes or tissues^12^.

A gene expression profile is an excellent next-generation biomarker for understanding cancer molecular diagnostics^13^. In this context, RNA-seq is a standard approach that compares the tumor gene expression profile to that of normal tissue to predict response to targeted therapies^14^. A study of single gene expression and its statistics, or the activation of molecular pathways, would provide insight into the mechanisms of cancer development, progression, and response to therapies^15^. Hence, next generation sequencing is currently crucial in the investigation of carcinogenesis and the identification of treatment targets, driver genes, drug targets, and biomarkers for human GC^16^.

The aim of the current study is to obtain the gene expression profile on PRU treated gastric cancer cells. A sequencing strategy using Illumina Novaseq6000 platform was adopted to acquire the differential genes among the control and PRU treated conditions which laid a foundation on understating the cell death mechanism. The overall schematic workflow of the present study is depicted in and as Fig. S1.

## Results

### Preprocessing of sequencing reads

AGS cells grouped into untreated and treated conditions with PRU for 24h (Fig. S2a) were sequenced using Ilumina novaseq6000 platform. Each condition were sequenced in triplicates. The sum of all the raw sequences were found to be 324,191,686 and the genome mapped sequences were 289,528,324, respectively. Samples from each conditions are mapped with average total reads 89.32% and average clean reads 95.98% as shown in Fig. S2b. The obtained cleaned reads were subjected to normalization based on log2 transcripts per kilo base million (TPM) (Fig. S2c). The samples were clustered based on Euclidean distance method and represented by dendogram as shown in Fig. S2d. The correlation of the sequenced samples is represented by heat map (Fig. S2e) based on read counts ≥5 and TPM ≥0.3, respectively. The overall read mapping statistics of all the samples are depicted in Table S1.

### Screening of differentially expressed genes

**Figure 1:**
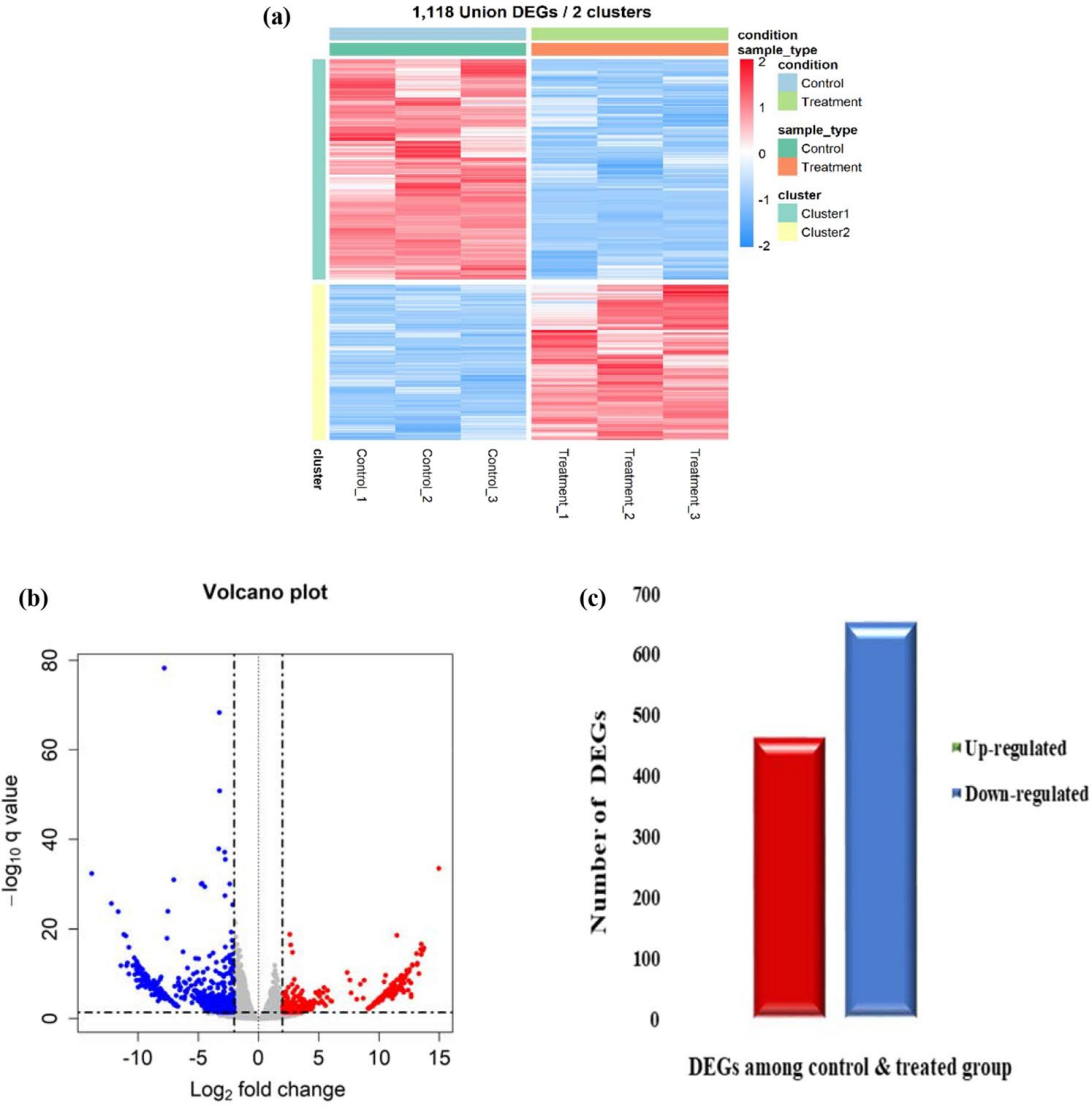
Identification of differentially expressed genes. (**a**) Heat map construction on the differentially expressed genes among control and treated conditions. (**b**) Volcano plot showing the differential pattern of genes in consideration of |logFC| >= 2, FDR < 0.05. (**c**) Bar diagram of total differentially expressed genes depicting the up-regulated and down-regulated categories.

The differentially expressed genes among PRU treated and untreated control AGS cells were identified using the edgeR package in R. The expression profile showed a total of 1,118 DEGs among the control and treated conditions which are clustered using complete linkage method as shown in Fig. 1a. Among the DEGs, the up-regulation and down-regulation category of genes were distinguished based on a logFC>=2 and false discovery rate (FDR) < 0.05 represented in a volcano plot (Fig. 1b). Upon identification, 463 genes were up-regulated and 655 were down-regulated as depicted in a bar graph in Fig. 1c.

### Functional enrichment of DEGs

**Figure 2:**
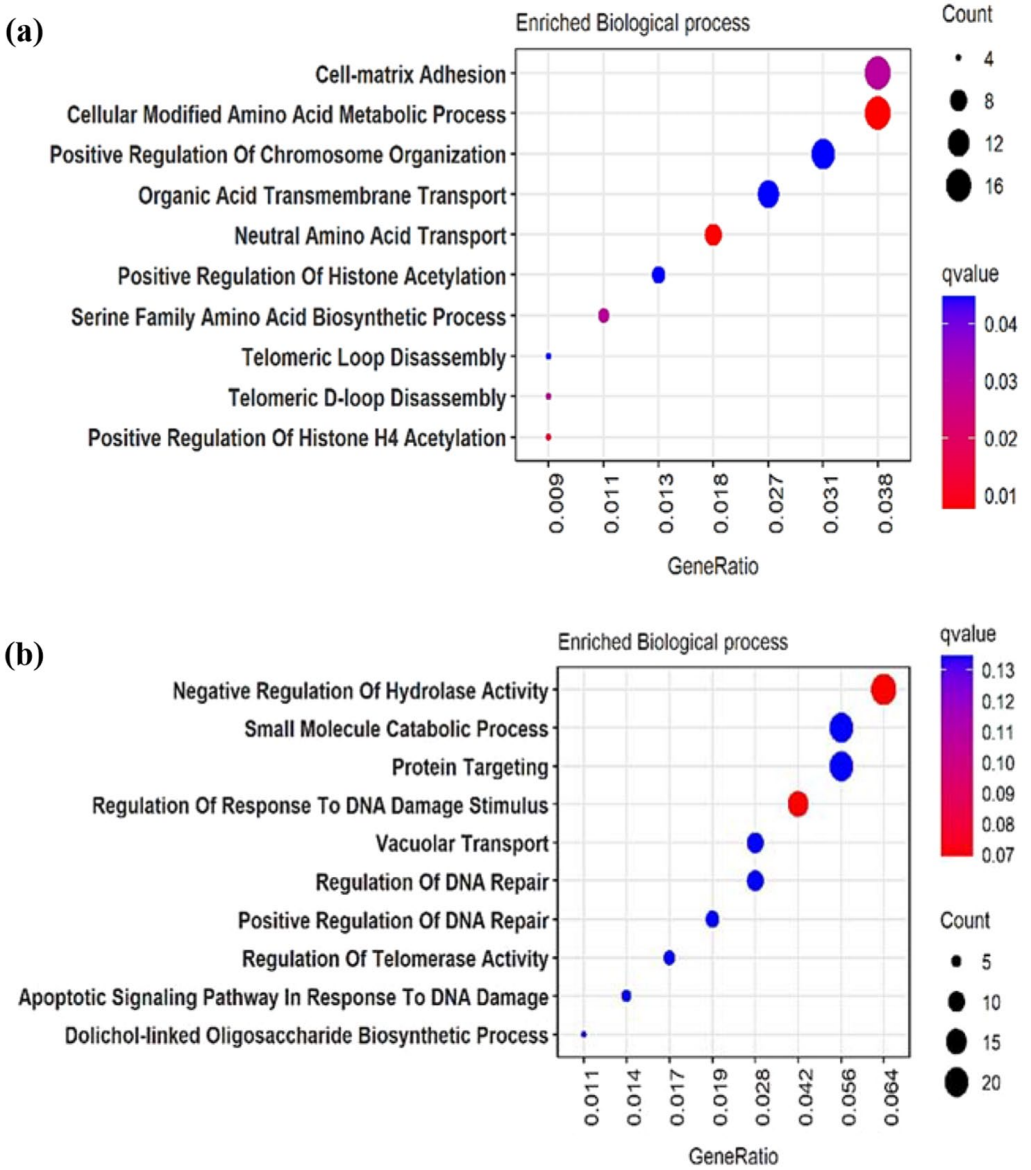
Gene ontology on the differentially expressed genes (DEGs). (**a**) Functionally enriched biological process of up-regulated genes. (**b**) Functionally enriched biological process of down-regulated genes.

The DEGs were subjected to functional enrichment analysis with the up-regulated and down-regulated cluster of genes using ClusterProfiler package in R. The gene ontology functions predicted within the q-value 2.0 were taken into consideration. The enriched biological processes of 463 up-regulated genes are found to be involved in cell matrix adhesion, cellular modified amino acids metabolic process followed by positive regulation of chromosome organization enriched in cell-matrix adhesion shown in Fig. 2a. Similarly, the 655 down-regulated genes were enriched in the categories of negative regulation of hydrolase activity, small molecule catabolic process, protein targeting followed by regulation of response to DNA damage stimulus shown in Fig. 2b.

### Pathway enrichment of DEGs

**Figure 3:**
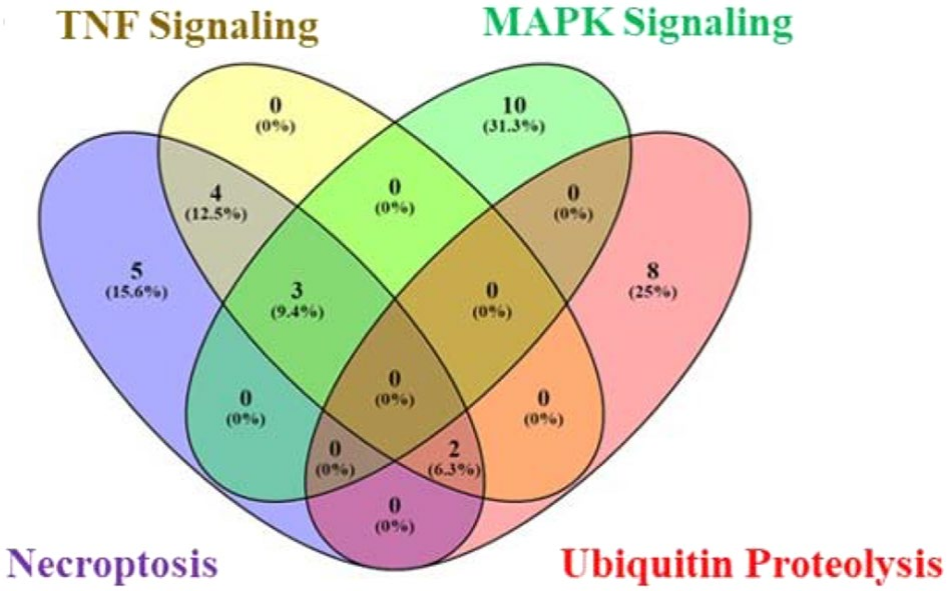
KEGG pathway enrichment on the DEGs. Venn diagram of top enriched KEGG pathways among the DEGs such as Necroptosis, TNF signaling, MAPK signaling and Ubiquitin proteolysis.

To obtain detailed information on the pathways executed by the DEGs, pathway enrichment was performed using KEGG database^44^. The top 10 significantly enriched pathways in DEGs with the number of genes along with FDR values are identified and listed in Table 1. Among the 10 pathways, maximum number of genes with higher FDR values were found to be involved in four distinct mechanisms: necroptosis, TNF signaling, MAPK signaling and Ubiquitin proteolysis represented in Fig. 3.

**Table 1:** List of top significantly enriched pathways among the DEGs.

Term ID	Pathway description	Gene count	FDR value
hsa04217	Necroptosis	12	7.38E-22
hsa04668	TNF signaling pathway	9	5.75E-16
hsa04010	MAPK signaling pathway	13	5.06E-12
hsa04120	Ubiquitin mediated proteolysis	10	4.96E-11
hsa04621	NOD-like receptor signaling pathway	10	2.63E-12
hsa04657	IL-17 signaling pathway	11	0.0001179
hsa05200	Pathways in cancer	12	1.56E-05
hsa01100	Metabolic pathways	13	1.11E-29
hsa04310	Wnt signaling pathway	6	0.0001296
hsa05205	Proteoglycans in cancer	7	0.0046199

### Expression of necroptosis related genes

**Figure 4:**
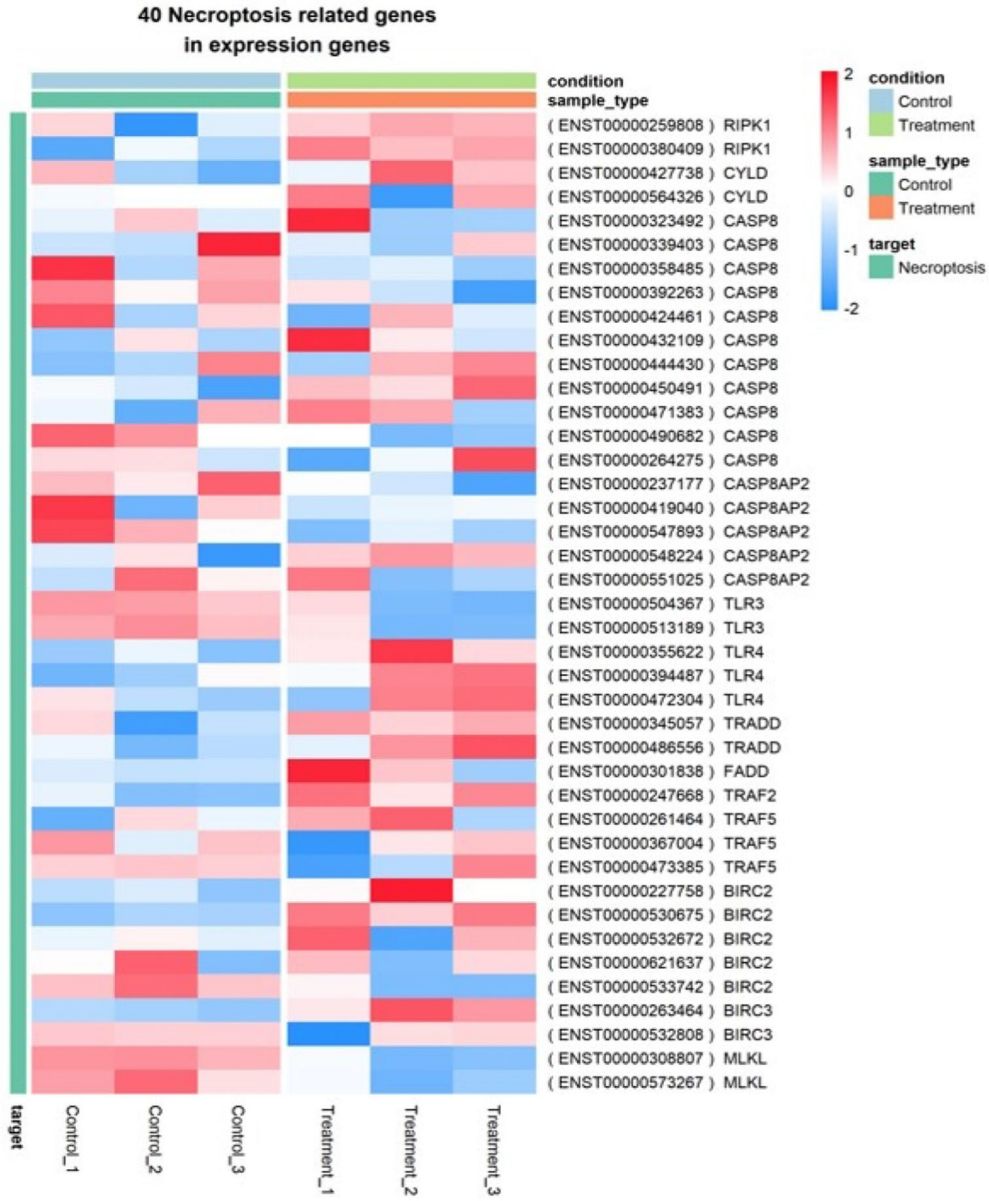
Necroptosis related genes among the DEGs. Heat map of list of necroptosis related genes expressed among DEGs.

**Figure 5:**
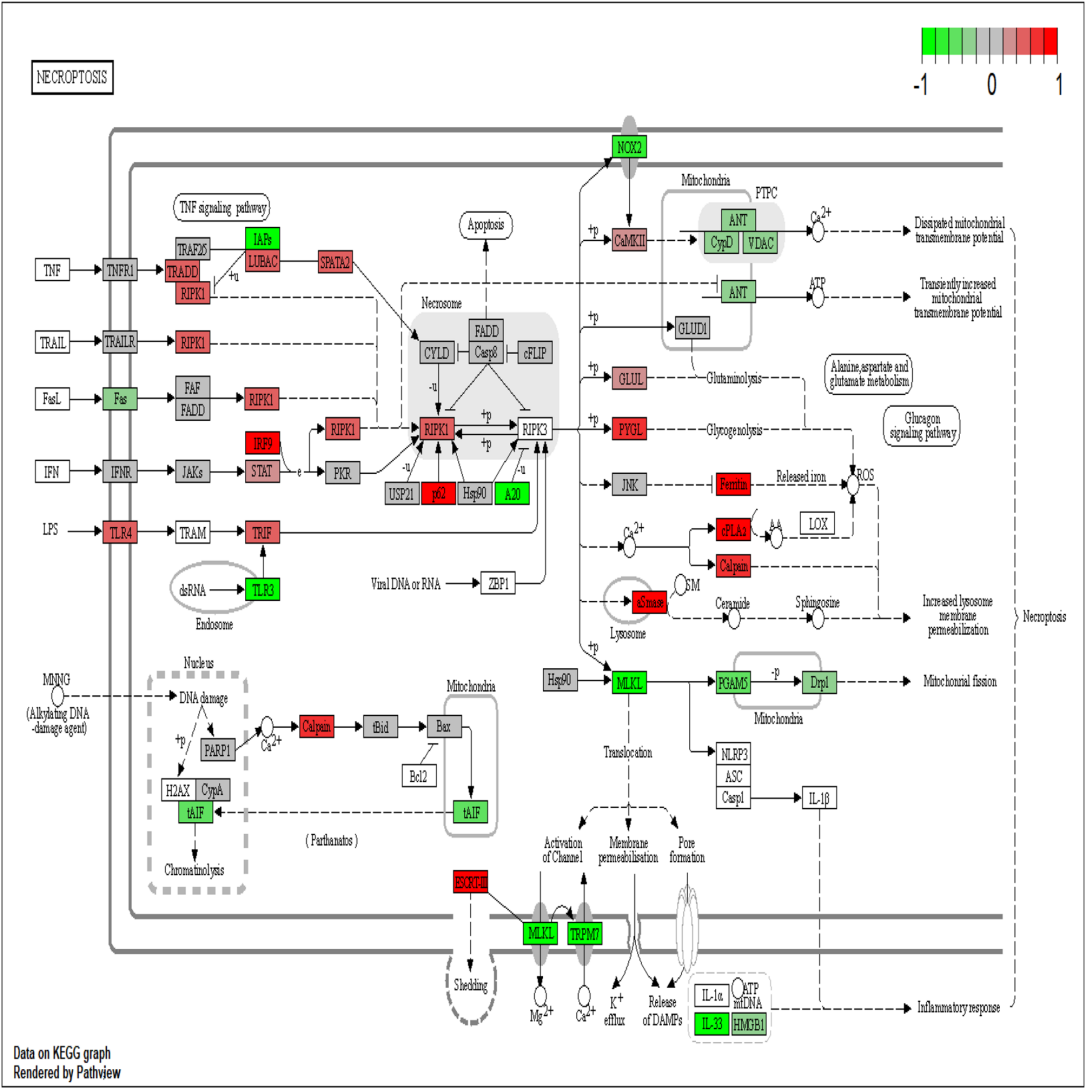
KEGG pathway construction on necroptosis. Path view mapper visualization of enriched genes using KEGG database in necroptosis pathway on Prunetin mediated cell death^44^ in AGS cells. (Multiple isoforms are used as average log fold change values).

With the significant pathway information involved in necroptosis signaling, the available necroptosis related genes were identified among the DEGs. Interestingly around 40 genes were found to be related with necroptosis as shown in Fig. 4. The enriched expression of genes involved in the necroptosis signaling network were mapped using KEGG pathway maps using Pathview R and visualized in Fig. 5.

### Functional annotation of the necroptosis genes

**Figure 6:**
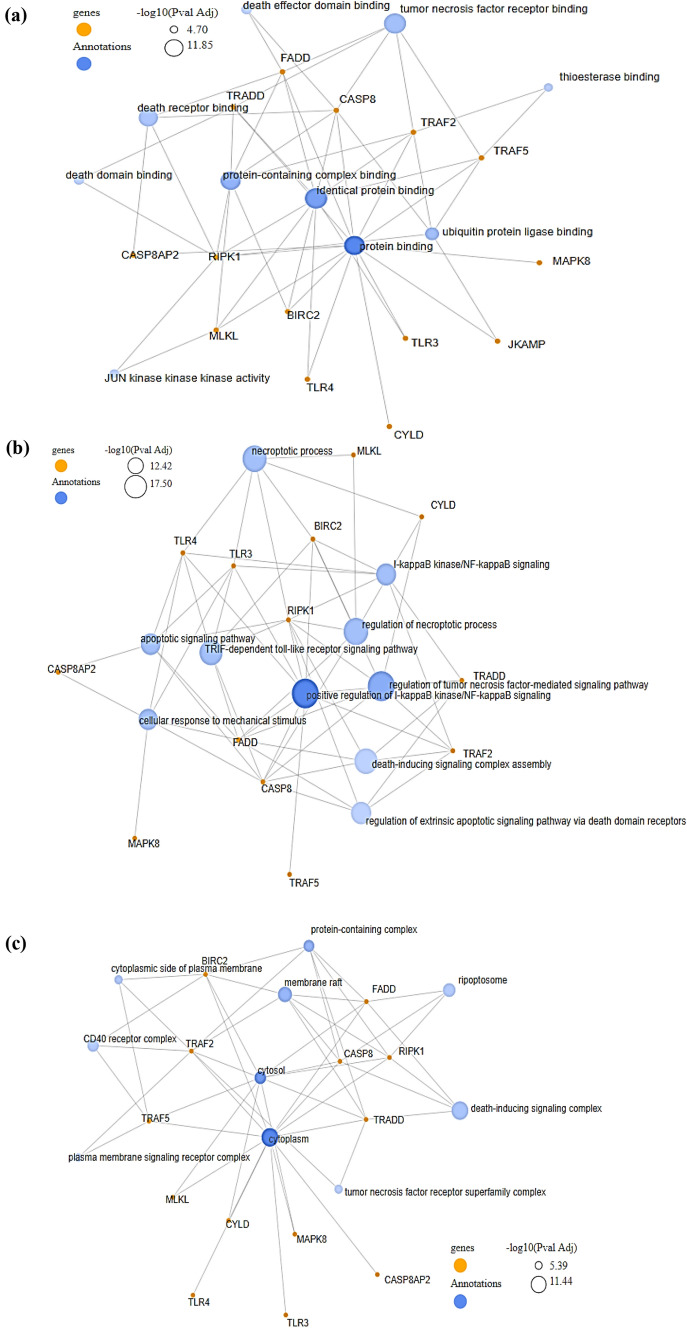
Network clusters of the significant necroptosis genes annotation in terms of (**a**) Biological process, (**b**) Molecular function, and (**c**) Cellular component.

The significant necropsis DEGs were subjected to functional analysis on GeneCodis. The top 10 GO terms of the necroptosis DEGs were listed and provided in Table S2-S4. Based on the constructed network clusters, the biological process of the genes were significantly enriched in identical protein binding (GO:0042802), tumor necrosis factor receptor binding (GO:0005164), protein binding (GO:0005515), protein-containing complex binding (GO:0044877), death receptor binding (GO:0005123), ubiquitin protein ligase binding (GO:0031625), death effector domain binding (GO:0035877), death domain binding (GO:0070513), JUN kinase kinase kinase activity (GO:0004706) and thioesterase binding (GO:0031996) shown in Fig. 6a.

Regarding MF, Regulation of tumor necrosis factor-mediated signaling pathway (GO:0010803), Positive regulation of I-kappaB kinase/NF-kappaB signaling (GO:0043123), Regulation of necroptosis process (GO:0060544), Necroptotic process (GO:0070266), Death-inducing signaling complex assembly (GO:0071550), TRIF-dependent toll-like receptor signaling pathway (GO:0035666), Regulation of extrinsic apoptotic signaling pathway via death domain receptors (GO:1902041), I-kappaB kinase/NF-kappaB signaling (GO:0007249), Apoptotic signaling pathway (GO:0097190) and Cellular response to mechanical stimulus (GO:0071260) shown in Fig. 6b.

Followed by, in terms of cellular component the genes were enriched in death-inducing signaling complex (GO:0031264), cytoplasm (GO:0005737), membrane raft (GO:0045121), ripoptosome (GO:0097342), cytosol (GO:0005829), CD40 receptor complex (GO:0035631), protein-containing complex (GO:0032991), tumor necrosis factor receptor superfamily complex (GO:0002947), cytoplasmic side of plasma membrane (GO:0009898) and plasma membrane signaling receptor complex (GO:0098802) shown in Fig. 6c.

### Identification of target gene and its associated prognostic markers

**Figure 7:**
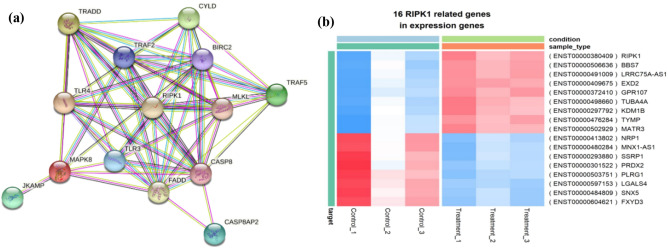
Hub gene identification and expression of its associated targets among DEGs. (**a**) Protein-protein interaction network of significant necroptosis DEGs (CYLD, TRADD, TRAF2, BIRC2, TLR4, RIPK1, CASP8, TRAF5, MAPK8, JKAMP, FADD, MLKL, CASP8AP2, and TLR3). (**b**) Heat map construction on the 16 candidate RIPK related gene expression. The differential expression of 16 RIPK genes were expressed between control and treated conditions based on fold change.

To determine the hub target gene among the significant necroptosis genes, a protein-protein interaction network was constructed using the STRING database with the threshold confident score of 0.9 and all the unconnected nodes were removed. Subsequently, on the observation of the PPI network, RIPK was found to be the core hub gene interconnected with all the necroptosis proteins (Fig. 7a). Among the total DEGs, 16 candidate genes were identified to be in close association with the target RIPK family of genes. In which, 9 genes (*RIPK1, EXD2, BBS7, LRRC75-AA51, GPR107, TUBA4A, KDM1B, TYMP,* and *MATR3*) were found to be up-regulated and 8 genes (*NRP1, MNX1AS1, SSRP1, PRDX2, PLRG1, LGALS4, SNX5* and *FXYD3*) were found to be down-regulated in PRU treated conditions presented in Fig. 7b, which can be considered as possible candidate biomarkers. The List of candidate genes significantly associated with RIPK family with their functional description is represented in Table S5.

### Candidate gene validation through GEPIA

Consistent with the GEO analysis, GEPIA analysis showed among the 16 candidate genes, 8 genes (*NRP1, MNX1-AS1, SSRP1, PRDX2, PLRG1, LGALS4, SNX5*, and *FXYD3*) were overexpressed in stomach cancer samples compared with normal tissues. The box plots of gene expression by pathological stages based on the TCGA clinical annotation revealed their high expression levels significantly associated with advanced adenocarcinoma stage (P-value<0.05) are shown in Fig. S3. In addition, the overall survival rate for the eight candidate genes were predicted with their HR and Logrank P ratios as follows: HR=0.68, P=0.017 (NRP1), HR=1.8, P=0.00019 (MNX1), HR=0.84, P=0.28 (SSRP1), HR=0.88, P= 0.42 (PRDX2), HR=1.1, P=0.55 (PLRG1), HR=0.82, P= 0.21 (LGALS4), HR=1.0, P=0.9 (SNX5) and HR=1.2, P=0.28 (FXYD3). The survival plots generated using GEPIA are provided in Fig. S4.

## Discussion

The global concern on GC requires significant treatment approaches along with clear understanding of the cellular mechanism. Previously reported transcriptomic studies on GC suggested important information on identification of prognostic biomarkers and drug targets^17^. However, in spite of various limitations, to achieve a full understanding of molecular mechanisms governing GC numerous alternative events at the isoform level is preferentially required^18^. The era of transcriptome profiling provides abundant opportunities to investigate thousands of genes, analyze complicated molecular mechanisms related to oncogenesis, and thereby contributes to the precision medicine^19^.

Our previous reported study on the molecular action of PRU represented an in vitro evidence on the induction of necroptosis cell death in gastric carcinoma cells^8^. In the present research, next generation sequencing platform was employed on the RNA samples of PRU treated AGS cells to get insight on the differential gene expression profile and identify the candidate biomarkers.

A total average of 48 million reads per sample were generated from RNA sequencing using pair-end Illumina Novaseq6000 platform with 89% mapping rate. Upon normalization, the low quality reads and adaptor reads were trimmed to obtain high stringency yield of 95.98% total clean reads on the reference genome GrCh38. Then, while comparing the normal and PRU treated AGS cells, a list of 1,118 DEGs were identified based on logFC >= 2, FDR < 0.05, respectively. Among all the genes, 463 were up-regulated and 655 were down-regulated. Biological enrichment on the up/down DEGs were shown to be highly involved in cell-matrix adhesion and negative regulation of hydrolase activity. Followed by pathway analysis revealed the involvement of crucial signaling mechanisms such as necroptosis, MAPK signaling, TNF signaling and ubiquitin proteolysis with significant gene ratio.

Based on our previous findings that Prunetin tends to activate necroptotic mediated cell death^8^, we decided to focus on necroptosis as the flavonoid's mode of action. Also, supporting research suggests that compounds have been shown to target necroptosis in gastric cancer^45,46^. Followed by, a list of 40 necroptotic genes that were differentiated among the treated and untreated conditions were identified. Among which *RIPK1* was observed and it is revealed to be associated closely with TNF mediated signaling that aids in the balance of cell death with interaction of TRADD/FADD receptors in the cell membrane localization^20^. Also, *RIPK1* is a prominent factor of necrosome complex formation along with RIPK3 and caspase 8 proteins during the execution of necroptosis^21,22^.

Further, a group of 15 genes (*EXD2, BBS7, LRRC75-AA51, GPR107, TUBA4A, KDM1B, TYMP, MATR3NRP1, MNX1AS1, SSRP1, PRDX2, PLRG1, LGALS4, SNX5* and *FXYD3*) were found to be in close association with the RIPK family. The interaction among the genes also showed RIPK1 to be at the central node and core hub of the network. As a validative approach, the genes were analyzed based on their expression profile using TCGA and GTEx data by GEPIA analysis. Specifically, 8 genes (*NRP1, MNX1AS1, SSRP1, PRDX2, PLRG1, LGALS4, SNX5* and *FXYD3*) were found to be highly expressed in stomach cancer tissues compared to normal tissues. Furthermore, these candidate genes have been shown to have elevated expression in gastric cancer samples as evidenced by high throughput sequencing and expression profiling studies. *NRP1* has been identified as a key regulatory axis in gastric cancer, as evidenced by sequencing (Reference: GSE192631). *MNX1-AS1, PLRG1*, and *SNX5* gene expression levels were found to be higher in 70 primary gastric cancer samples after genome-wide expression profiling. (Reference: GSE35809). Gene expression profiling using oligonucleotide arrays on 22 gastric cancer tissues revealed an increase in the expression of the genes *LGALS4, FXYD3, SSRP1*, and *PRDX2* (Reference: GSE2685).

*NRP1* gene that encodes for neuropilin that are involved in nervous system development and is reported to be an important factor in the *VGEF* cell differentiation during angiogenesis process^23,24^. The transcription regulatory gene *MNX1AS1* plays a vital role in methylation process during epigenetic activation and reported to contribute in gastric cancer progression^25,26^. *SSRP1* is known as a component of chromatin formation complex that acts as a transcriptional cofactor for p63/TP53. *SSRP1* is identified to bind specifically to the double strand of DNA in combination with anti-tumor agents to execute cell death by blocking replication^27^. Furthermore, peroxiredoxin enzyme protein and pleiotropic regulator enzyme encoded by *PRDX2* and *PLRG1* genes has been involved directly in mRNA splicing during the spliceosome complex formation^28,29^. Galectin-4 protein encoded by the gene *LGALS4* is implicated in the assembly of cell junction and adhesion. Also, the protein is reported to be an assessment blood marker factor employed in the screening of colorectal cancer^30^. *SNX5* gene encodes for intracellular membrane protein receptors that interacts in cellular trafficking during the calcium/potassium ion channel transport and endocytosis. Interestingly, increased expression of *SNX5* has been evidenced in the progress of cancer cell proliferation through *EGFR*-*ERK1*/*2* signaling pathway^31^. Similarly, *FXYD3* is noted to show overexpression in carcinoma cells with extensive role in the cytoplasm during the tumorigenesis development^32,33^. Interestingly, our study has shown that all these significant genes are found to be down-regulated in their expression in PRU-treated conditions.

As a therapeutic agent, a compound should induce higher expression of the target gene in proportion to shorter survival^47^. In this regard, our survival analysis of the candidate genes revealed a shorter survival degree with increased expression, with the exception of *MNX1* and *FXYD*3. The identification of specific molecular phenotypes has significant implications for treatment strategies and ongoing drug development^48^. Herein, to shed further light on gastric cancer tumorigenesis, we presented the presence of candidate gene expression as a set of supporting evidence showing the different molecular subtypes of GC: (Epstein–Barr virus (EBV), microsatellite instability (MSI), genomically stable (GS), and chromosomal instability (CIN) using cBioportal database. The implications of molecular phenotype would be significant, and it would imply the presence of distinct molecular drivers and molecular pathways for each gastric cancer subtype to therapy^49^.

The present transcriptome study on AGS cells treated with PRU was undertaken to explore the whole genome level on the same cell line based on our previous study that demonstrated the activation of necroptosis mechanism by PRU^8^. The research disclosed DEGs with 16 RIPK family of genes that were associated with DNA damage, cell adhesion, differentiation, angiogenesis and transcription regulation. Among which, *NRP1, MNX1, SSRP1, PRDX2, PLRG1, LGALS4, SNX5* and *FXYD3* were identified as candidate biomarkers validated through GEPIA analysis. These 8 genes may be used as putative markers against gastric cancer upon further validations in the induction of cell death by PRU. Although cell lines are preliminary models for studying the tumor environment, they lack the ability to closely mimic the in vivo environment which is a limitation of the current research. In this context, extended investigation in multiple cell lines for versatility and in vivo model validation is still required in future.

## Conclusion

In conclusion, the genomic data obtained could be used to elucidate and generate hypotheses on the mechanisms of action of PRU in AGS cells. The identified differential gene expression pattern provides insight on the involvement of PRU mediated cancer cell death found to be in close association with RIPK family genes through necroptosis mechanism. Specifically, 8 genes *NRP1, MNX1, SSRP1, PRDX2, PLRG1, LGALS4, SNX5* and *FXYD3*) which are highly expressed in stomach cancer were significantly down-regulated in PRU treated samples. This transcriptome study, combined with gene expression analysis, suggests that these eight genes could be used to identify new biomarkers for the treatment of GC.

## Methods

### Cell culture and cultivation

The human gastric cancer cell lines AGS was obtained from the Korea cell line bank (Seoul, Korea). Upon arrival, AGS cells were cultured in Roswell Park Memorial Institute (RPMI)1640 medium supplemented with 10% heat inactivated fetal bovine serum (FBS); 100 U/mL penicillin and 100 μg/mL streptomycin was used as antibiotics. The cultivation conditions of the cells were maintained at 37 °C in a humidified atmosphere of 95% air and 5% CO_2_ in a 100 mm petri dish.

### Prunetin treatment

AGS cells were allowed to grow up to 70%-80% confluency rate in consideration for experimentation. Upon attaining 80% growth, AGS cells were digested using trypsin at 37 °C for 4 min. Followed by dilution of trypsin was performed by adding fresh medium. The cell culture was subjected to centrifugation at 1200 rpm for 4mins, then the cell pellet was suspended in fresh medium. The obtained cells were counted using a hemocytometer and seeded onto 60 π plate with a seeding density of 4 X 10^4^/well and incubated for 24 h.

### Isolation of RNA for sequencing

After 24h treatment, the whole cell lysates were collected and washed with 1X PBS (for two times). The total RNA was extracted individually from three samples of control group and three samples of treatment group, using TRIzol reagent (Invitrogen Thermofischer Scientific). The total RNA isolated was suspended in diethylpyrocarbonate (DEPC) (iNtRON Biotechnology). The integrity of RNA was then quantified using Nanodrop spectrophotometer (Thermo Scientific) and quality-assessed by RNA 6000 Nano assay kit (Agilent) and Bioanalyser2100 (Agilent).

### Library construction and sequencing

To obtain high-throughput transcriptome data of Human, we implemented Illumina-based NGS sequencing. NGS sequencing libraries were generated from one microgram of total RNA using TruSeq RNA Sample Prep Kit (Illumina) according to the manufacturer's protocol. In brief, the poly-A containing RNA molecules were purified using poly-T oligo attached magnetic beads. After purification, the total poly A+RNA was fragmented into small pieces using divalent cations under elevated temperature. The cleaved mRNA fragments were reverse transcribed into first strand cDNA using random primers. Short fragments were purified with a QiaQuick PCR extraction kit and resolved with EB buffer for end reparation and addition of poly (A). Subsequently, the short fragments were connected with sequencing adapters. Each library was separated by adjoining distinct MID tag. The resulting cDNA libraries were then paired-end sequenced (2x101bp) for samples with Novaseq 6000 system (Illumina).

### Identification of differential gene expression

Paired end sequence files from six samples (Fastq: R1, R2) were obtained and subjected to processing using Trimmomatic −0.36^34^ with parameter settings like leading:5, trailing:5, sliding window:4:15, and minlen:36. After quality score checking and read length checking RNASeq reads were mapped to human reference genome GRCh38^35^ (Gencode release 12) using STAR^36^ with default parameters. Accurately quantifying the expression level of a gene from RNASeq reads was identified by using RSEM^37^. RSEM assembles individual transcripts from RNASeq reads that have been aligned to the genome sequences. And then TPM was calculated with each transcribed fragments in the sample to quantify the expression level. To analyze the gene expression based on the transcripts, the genes where all samples in any condition had more than 5 NumRead and more than 0.3 TPM were counted as expressed genes and included in following analyses. To compare with each samples, TPM were conducted Global normalization and were used for further analysis.

### Gene ontology and KEGG pathway analysis

The normalized expression profiles of the DEGs expressing more than 0.3 TPM and 5 read counts were used for DEGs analysis using edgeR v3.22.5^38^. Any expressed genes of which log2-fold-change value was more than two and false discovery rate (FDR) was under 0.05 between any comparative set of two of conditions were selected as DEGs. The expression profile of each gene was hierarchically clustered by complete linkage method. The visualization was implemented using ggplot2 library in R packages. Differentially expressed gene sets were functionally enriched based on gene ontologies (GOs) and KEGG orthologies (KOs) using ClusterProfiler R package v3.16.1^39^. The only enriched functions with under the q-value of 0.2 were counted. Enriched KOs were mapped on KEGG pathway maps using Pathview R package v1.28.1^40,41^. Then we constructed the binary heatmap showing all genes involved in the significant pathways using in-house R script.

### Functional analysis and Protein–protein interaction (PPI) network construction of the candidate genes

The candidate genes related to RIPK family was subjected to functional enrichment using Genecodis^42^. The genes were functionally annotated with their geneIDs and significant network clusters on each category (biological process, molecular function & cellular component) was constructed. Also, protein–protein interaction network was performed uisng the Search Tool for the Retrieval of Interacting Genes (STRING) (https://string-db.org/) to identify the hub gene. The confidence score for the construction of an interactive network was set up with a threshold of 0.9, respectively.

### Target gene expression analysis by GEPIA

The Gene Expression Profiling Interactive Analysis (GEPIA) database (http://gepia.cancer-pku.cn/) is a web based tool to deliver fast and customizable functionalities based on The Cancer Genome Atlas (TCGA) and Genotype-Tissue Expression (GTEx) data^43^. The identified 16 RIPK genes were validated by the association of their gene expression levels with STAD (stomach adenocarcinoma) tissue and normal stomach tissues. The statistically range was adopted using *P* < 0.05 with a fold change of >2 as a descriptive threshold.

## Supplementary Information

Supplementary Information.

## Data Availability

The datasets generated during the current study are available in the GEO database with the accession no. GSE198930 [https://www.ncbi.nlm.nih.gov/geo/query/acc.cgi?acc=GSE198930].
