# Supplementary material for: Investigation on the cellular mechanism of Prunetin evidenced through next generation sequencing and bioinformatic approaches against gastric cancer
Source: Sci Rep. 2022 Jul 13;12:11852. doi: 10.1038/s41598-022-15826-y (PMC9279440; doi:10.1038/s41598-022-15826-y)
Supplement: Supplementary file 1 — Supplementary Information. [file 41598_2022_15826_MOESM1_ESM.pdf]

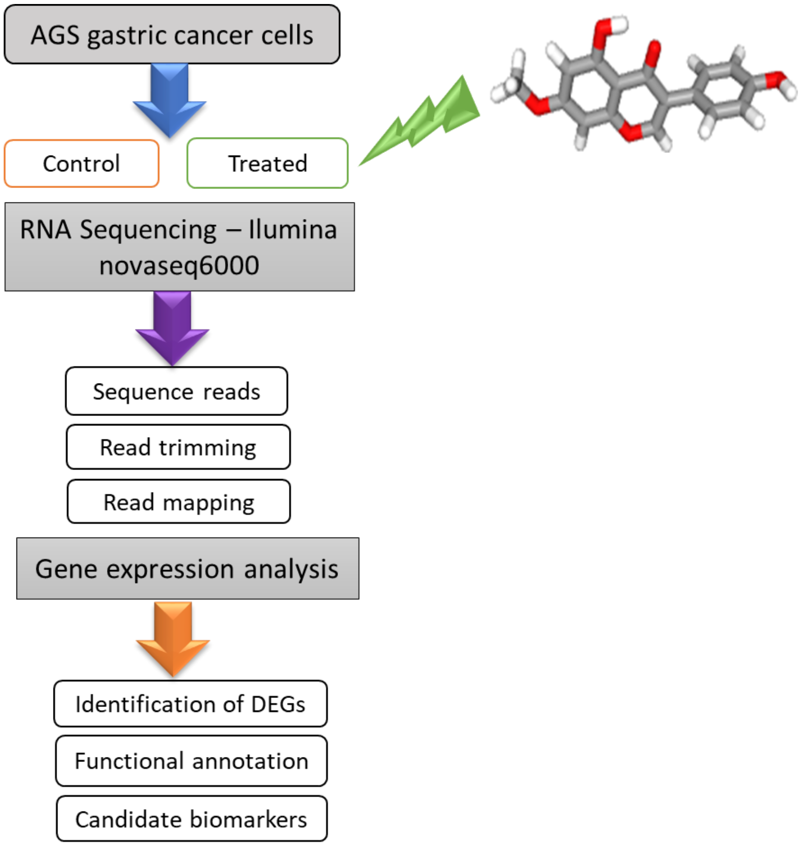

Fig. S1. Comprehensive workflow of transcriptome profiling for the identification of candidate biomarkers on prunetin against gastric cancer.

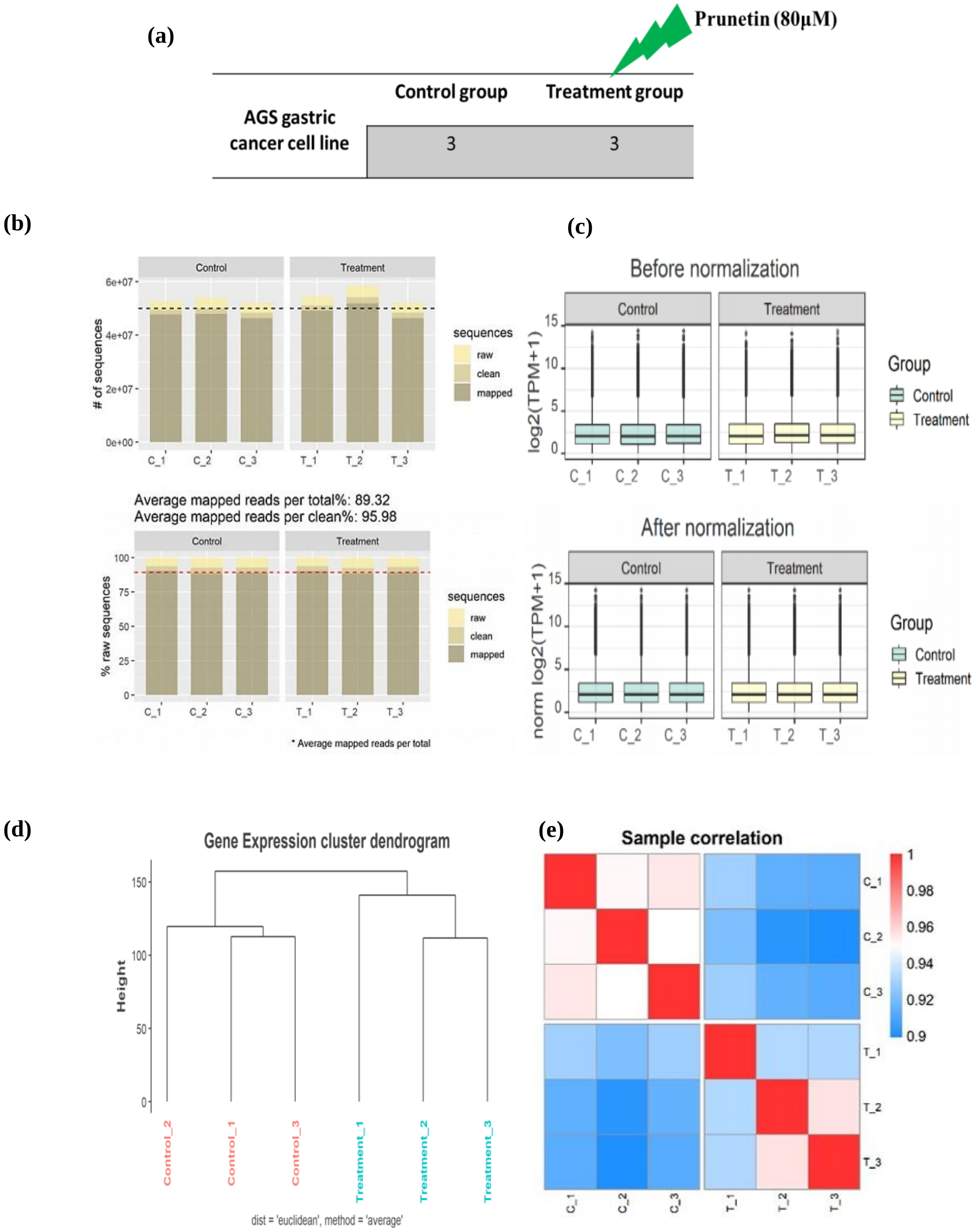

Fig. S2. Sequencing statistics and preprocessing of reads. **(a)** AGS cells were distinguished in two groups as control/untreated (0 µM) and treated with PRU (80 µ M). **(b)** Bar diagram of average raw reads mapped to obtain total clean reads. **(c)** Normalization of total reads in control and treatment groups based on log2 (TPM). **(d)** Dendogram of gene expression clustered based in Euclidean distance method**.** **(e)** Heat map showing the correlation of the samples in two conditions. (Read counts ≥5, TPM ≥0.3 were considered at least in one condition)

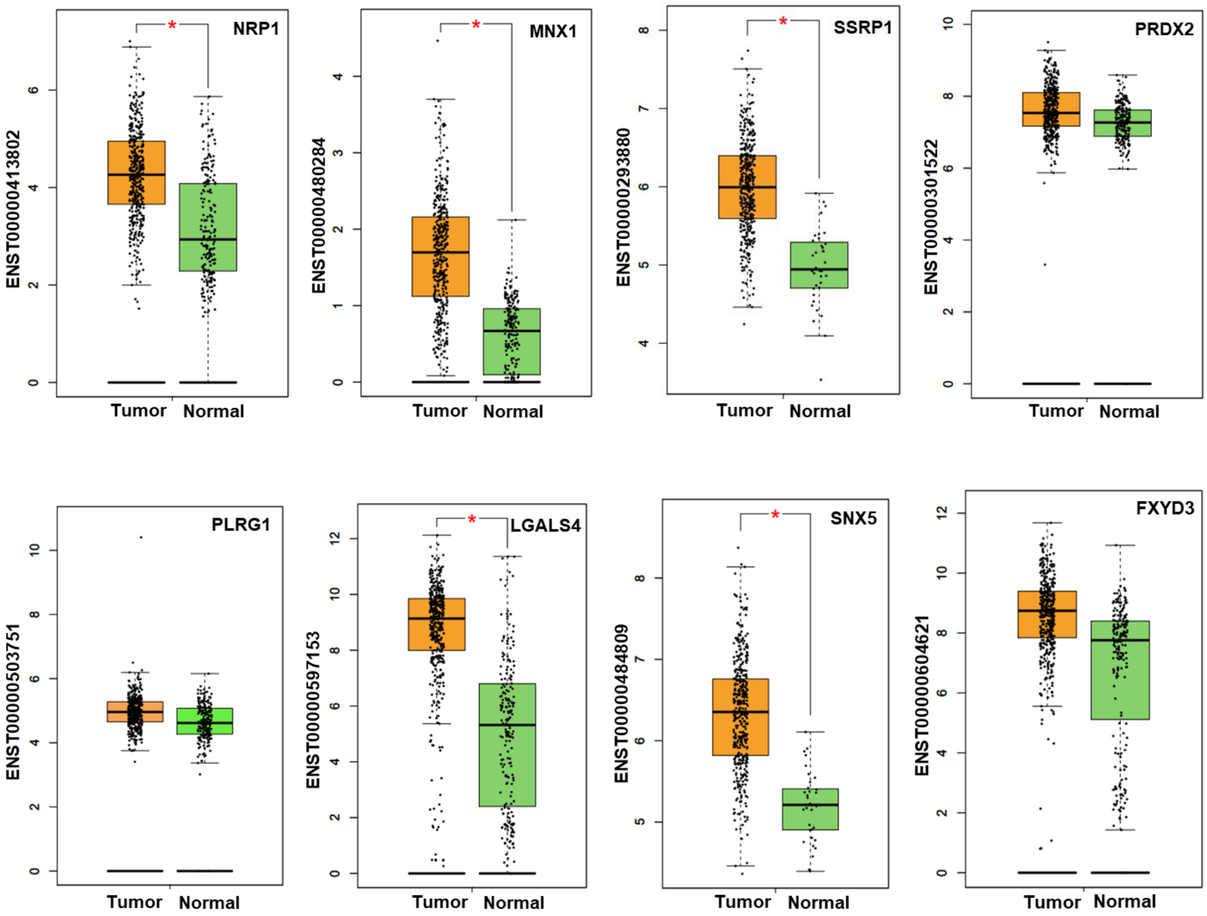

Fig. S3. Validation of the candidate genes. Boxplots of *NRP1, MNX1, SSRP1, PRDX2, PLRG1, LGALS4, SNX5* and *FXYD3* genes represented based on TCGA and GTEx data in GEPIA. The orange and green boxes represents STAD tissues and normal tissues, respectively. [STAD: Stomach adenocarcinoma].

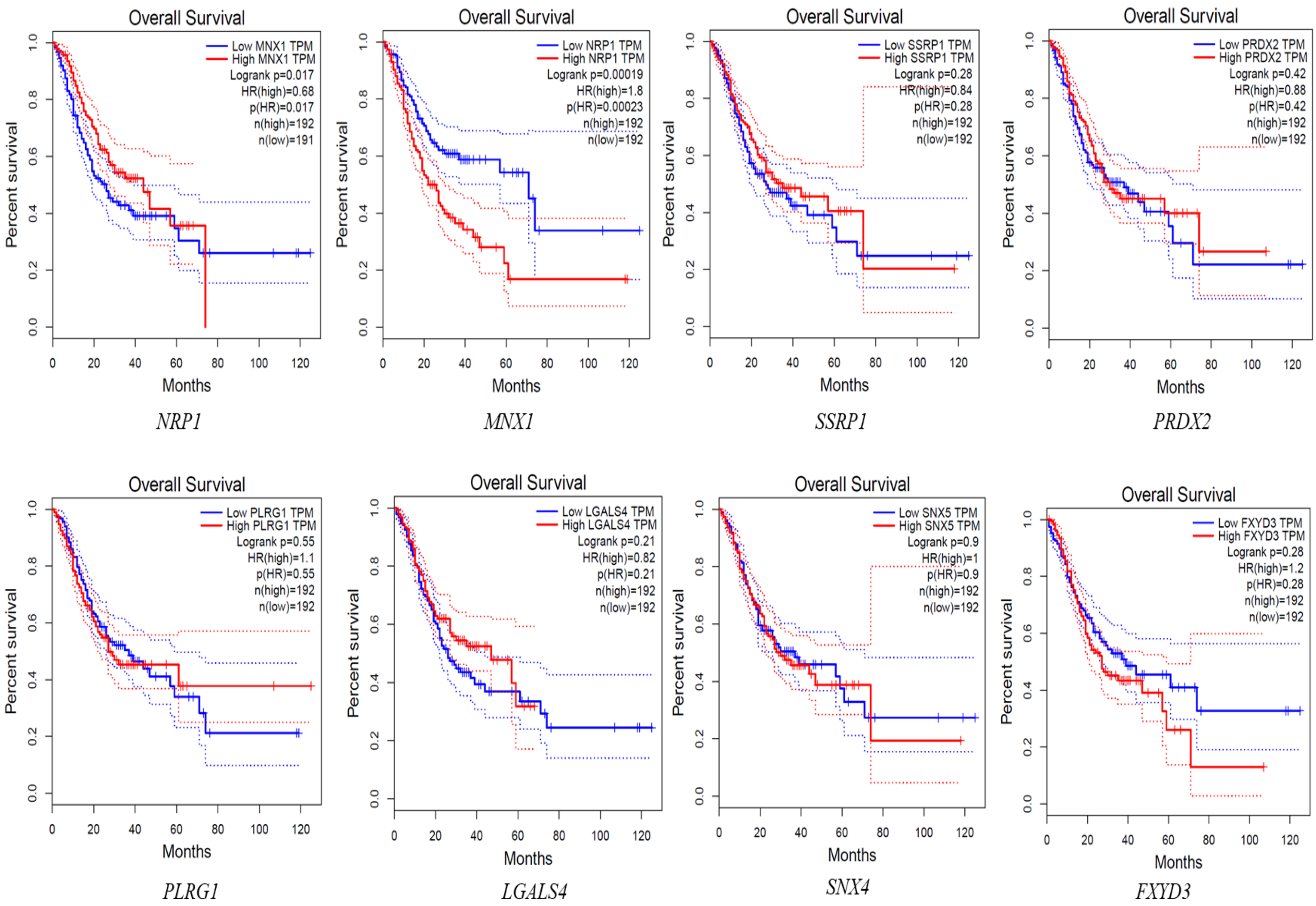

Fig. S4. Survival analysis of the candidate genes through GIEPA. Survival plot for the selected candidate genes were plotted through GIEPA analysis using TCGA datasets on Stomach adenocarcinoma (STAD).

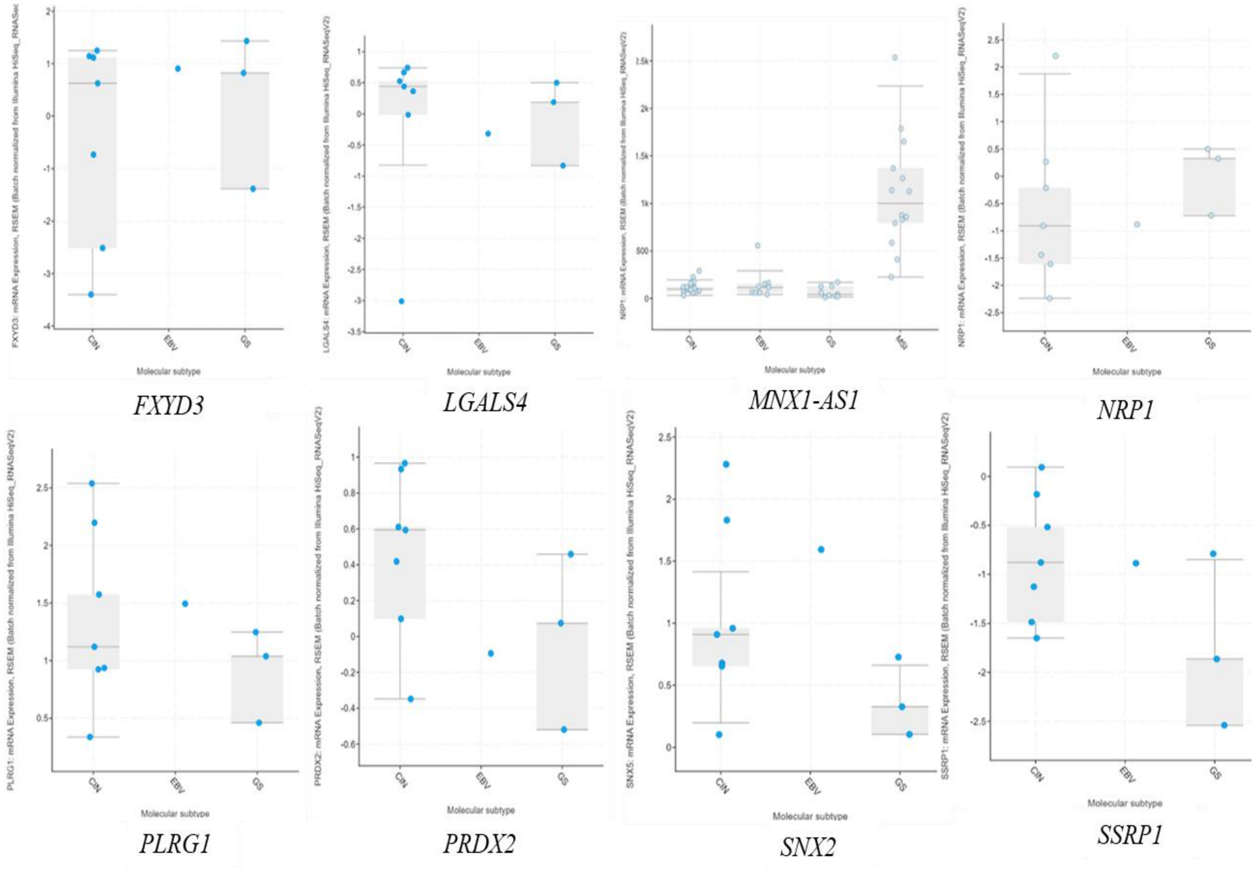

Fig. S5. Expression of the candidate genes in different GC molecular subtypes using cBioportal. Box plot representation of the candidate genes in different molecular subtypes (Epstein Barr virus-EBV, microsatellite instability-MSI, genomically stable-GS, and chromosomal instability-CIN).

Table S1a. Preprocessed read counts

(a)

Sample	Raw sequences	Raw nucleotides	Clean_sequences	Clean_nucleotides
C_1	52,788,970	5,331,685,970	49,493,720	4,972,141,207
C_2	54,096,752	5,463,771,952	50,111,546	5,034,108,278
C_3	52,107,172	5,262,824,372	48,376,722	4,860,402,564
T_1	54,580,764	5,512,657,164	51,097,056	5,133,653,011
T_2	58,622,196	5,920,841,796	54,184,500	5,442,985,130
T_3	51,995,832	5,251,579,032	48,396,794	4,861,280,841
**Sums**	324,191,686	32,743,360,286	301,660,338	30,304,571,031
**Average**	54,031,947.67	5,457,226,714.33	50,276,723	5,050,761,838.50

Table S1b. Read mapping statistics

(b)

Sample	Clean_sequences (%)	Clean_nucleotides (%)	Genome_mapped sequences	Mapping rate (%)
C_1	93.76	93.26	47,740,968	90.44
C_2	92.63	92.14	47,899,304	88.54
C_3	92.84	92.35	46,375,358	89
T_1	93.62	93.12	49,249,600	90.23
T_2	92.43	91.93	51,872,918	88.49
T_3	93.08	92.57	46,390,176	89.22
**Sums**	-	-	289,528,324	-
**Average**	93.06	92.56	48,254,720.67	89.32

Table S2. Top 10 significant GO terms of necroptosis DEGs in terms of cellular component

**Significant GO terms**	**Description**	**P value**	**Associated genes**
GO:0031264	death-inducing signaling complex	3.60E-12	*RIPK1,FADD,CASP8,TRADD*
GO:0005737	cytoplasm	3.96E-12	*RIPK1,TRAF2,CYLD,BIRC2,MLKL,TLR4,FADD, CASP8,TLR3,TRADD,MAPK8,TRAF5,CASP8AP2*
GO:0045121	membrane raft	2.84E-10	*RIPK1,TRAF2,BIRC2,FADD,CASP8,TRADD*
GO:0097342	ripoptosome	2.88E-09	*RIPK1,FADD,CASP8*
GO:0005829	cytosol	1.40E-08	*RIPK1,TRAF2,CYLD,BIRC2,MLKL,FADD,CASP8, TRADD,MAPK8,TRAF5*
GO:0035631	CD40 receptor complex	1.59E-08	*TRAF2,BIRC2,TRAF5*
GO:0032991	protein-containing complex	3.63E-08	*RIPK1,TRAF2,BIRC2,FADD,CASP8,TRADD*
GO:0002947	tumor necrosis factor receptor superfamily complex	1.98E-06	*TRAF2,TRADD*
GO:0009898	cytoplasmic side of plasma membrane	1.99E-06	*TRAF2,BIRC2,TRAF5*
GO:0098802	plasma membrane signaling receptor complex	4.04E-06	*TRAF2,TRAF5*

Table S3. Top 10 significant GO terms of necroptosis DEGs in terms of molecular function

Significant GO terms	Description	P value	Associated genes
GO:0010803	Regulation of tumor necrosis factor-mediated signaling pathway	3.13E-18	*RIPK1,TRAF2,CYLD,BIRC2,FADD, CASP8,TRADD*
GO:0043123	Positive regulation of I-kappaB kinase/NF-kappaB signaling	5.07E-18	*RIPK1,TRAF2,BIRC2,TLR4,FADD, CASP8,TLR3,TRADD,TRAF5*
GO:0060544	Regulation of necroptosis process	1.07E-16	*RIPK1,CYLD,BIRC2,MLKL,FADD, CASP8*
GO:0070266	Necroptotic process	2.22E-16	*RIPK1,CYLD,BIRC2,MLKL,TLR4, TLR3*
GO:0071550	Death-inducing signaling complex assembly	9.13E-16	*RIPK1,TRAF2,FADD,CASP8,TRADD*
GO:0035666	TRIF-dependent toll-like receptor signaling pathway	1.17E-15	*RIPK1,BIRC2,TLR4,FADD,CASP8, TLR3*
GO:1902041	Regulation of extrinsic apoptotic signaling pathway via death domain receptors	7.20E-14	*RIPK1,TRAF2,FADD,CASP8,TRADD*
GO:0007249	I-kappaB kinase/NF-kappaB signaling	1.11E-13	*RIPK1,TRAF2,BIRC2,TLR4,TLR3, TRADD*
GO:0097190	Apoptotic signaling pathway	1.88E-13	*RIPK1,TLR4,FADD,CASP8,TLR3, CASP8AP2*
GO:0071260	Cellular response to mechanical stimulus	3.83E-13	*TLR4,FADD,CASP8,TLR3,MAPK8, CASP8AP2*

Table S4. Top 10 significant GO terms of necroptosis DEGs in terms of biological process

Significant GO terms	Description	P value	Associated genes
GO:0042802	identical protein binding	1.43E-12	*RIPK1,TRAF2,BIRC2,MLKL,TLR4, FADD,CASP8,TLR3,TRADD,TRAF5*
GO:0005164	tumor necrosis factor receptor binding	1.65E-12	*TRAF2,FADD,CASP8,TRADD,TRAF5*
GO:0005515	protein binding	9.27E-12	*RIPK1,TRAF2,CYLD,BIRC2,JKAMP, MLKL,TLR4,FADD,CASP8,TLR3, TRADD,MAPK8,TRAF5,CASP8AP2*
GO:0044877	protein-containing complex binding	1.86E-11	*RIPK1,TRAF2,BIRC2,MLKL,FADD, CASP8,TRADD*
GO:0005123	death receptor binding	5.99E-11	*RIPK1,FADD,CASP8,CASP8AP2*
GO:0031625	ubiquitin protein ligase binding	4.32E-08	*RIPK1,TRAF2,JKAMP,CASP8,TRAF5*
GO:0035877	death effector domain binding	3.12E-06	*FADD,CASP8*
GO:0070513	death domain binding	4.54E-06	*RIPK1,TRADD*
GO:0004706	JUN kinase kinase kinase activity	1.13E-05	*RIPK1,MLKL*
GO:0031996	thioesterase binding	2.00E-05	*TRAF2,TRAF5*

Table S5. List of candidate genes significantly associated with RIPK family among the DEGs

S.No	Gene Name	Control	Treated	Up/Down	Function
1	BBS7	6.7	9.7	↑	Cilium biogenesis/degradation, Protein transport
2	LRRC75A-AS1	5.3	8.9	↑	Involved in the assembly of adherens junction
3	EXD2	3.7	9.2	↑	DNA damage and repair mechanism
4	GPR107	12.7	16.4	↑	Clathrin-dependent endocytosis
5	TUBA4A	1.7	4.5	↑	Regulation of G2/M transition of mitotic cell cycle
6	KDM1B	4.3	5.2	↑	DNA methylation, Regulation of transcription
7	TYMP	0.8	1.6	↑	Angiogenesis, Chemotaxis, Differentiation
8	MATR3	1.3	4.6	↑	Interaction with nuclear matrix proteins
9	NRP1	3.7	1.5	↓	Neurogenesis, Host-virus infection
10	MNX1-AS1	3.9	2.5	↓	T-box transcription factors, Wnt signaling
11	SSRP1	1.7	1.5	↓	Transcription regulation, Host-virus infection
12	PRDX2	18	14	↓	MAPK activation, Negative regulation of apoptosis
13	PLRG1	6.3	4	↓	mRNA processing & splicing
14	LGALS4	1	0	↓	Cell adhesion
15	SNX5	7.5	5	↓	Endocytosis, Protein transport
16	FXYD3	12	9	↓	Sodium/potassium ion transport
